# The complete mitochondrial genome of the *Hipposideros pendleburyi* (Pendlebury's leaf-nosed bat) an endemic species in Thailand

**DOI:** 10.1080/23802359.2021.2005493

**Published:** 2021-12-10

**Authors:** Wasitthee Kongkachana, Chaiwat Naktang, Duangjai Sangsrakru, Nukoon Jomchai, Phuset Yingyong, Wirulda Pootakham, Sithichoke Tangphatsornruang, Pipat Soisook

**Affiliations:** a National Omics Center, National Science and Technology Development Agency, Khlong Luang, Thailand; b Princess Maha Chakri Sirindhorn Natural History Museum, Prince of Songkla University, Hat Yai, Thailand

**Keywords:** *Hipposideros pendleburyi*, Pendlebury's Leaf-nosed bat, mitochondrial genome

## Abstract

This study presents the first complete mitochondrial genome of the *Hipposideros pendleburyi* (Pendlebury's leaf-nosed bat), an endemic species in Thailand. The mitochondrial genome was 16,820 bp in length and contains 13 protein-coding genes, 2 ribosomal RNA genes, 22 transfer RNA genes, and a control region. The overall base composition was 31.5% A, 26.2% T, 28.3% C, and 14.0% G. A maximum-likelihood tree revealed that *H. pendleburyi* was grouped with *Hipposideros armiger* within the Hipposideridae clade, which has Rhinolophidae as a sister clade.

The Pendlebury's leaf-nosed bat (*Hipposideros pendleburyi*) was named by Chasen in 1936 but had long been included in *Hipposideros turpis* (Lekagul and McNeely 1977; Corbet and Hill 1992; Francis 2008). Only a decade ago, it has been regarded as a distinct species and known only from peninsular Thailand (Soisook 2011; Thong et al. 2012). This species was found in limestone areas of seven provinces in peninsular Thailand with a colony size of up to 800 individuals (Soisook 2019). It has been assessed as a vulnerable species on the Red List of Threatened Species by IUCN, and its population size has continuously declined due to habitat disturbance by human activities and limestone quarrying (Soisook 2019). Until now, there is only one complete mitochondrial genome (*Hipposideros armiger*) of the bat in the family Hipposideridae available (Dong et al. 2017). The complete mitochondrial genome of *H. pendleburyi* provides valuable information for inferring the phylogenetic relationships of Chiroptera order and a foundation for future research.

A male specimen of *H. pendleburyi* was collected from Tham Le Stegodon Cave, Palian District, Trang Province, Thailand (7.141 N, 99.789 E). Bat sampling in this study was permitted by the Department of National Park, Wildlife and Plant Conservation (project number 6210306). Collection and handling of bats followed the guidelines of the American Society of Mammalogists (Sikes 2016). The specimen was deposited in the Mammal Collection of the Princess Maha Chakri Sirindhorn Natural History Museum, Prince of Songkla University (PSU), Hat Yai, Songkhla, Thailand (http://www.biology.sci.psu.ac.th/pipat-soisook/, Pipat Soisook: pipat.s@psu.ac.th) under the voucher number PSUZC-MM.2021.6. Total DNA was extracted using QIAamp Tissue Kit (Qiagen, Germany). A DNA sequencing library was constructed and paired-end reads (150 bp) were sequenced by Illumina HiSeqX Ten sequencer (Illumina, Singapore).

The *H. pendleburyi* mitochondrial genome was assembled *de novo* using MitoZ 2.4 (Meng et al. 2019) and annotated using the MITOS web server (Bernt et al. 2013). Protein-coding genes (PCGs) and RNA genes were confirmed using the Basic Local Alignment Search Tool (BLAST) (Altschul et al. 1990). The complete mitochondrial genome (GenBank Accession Number: MZ196220.1) was 16,820 bp in length including 13 PCGs, 2 ribosomal RNA genes (rRNAs), 22 transfer RNA genes (tRNAs), and a non-coding control region. The overall base composition was 31.5% A, 26.2% T, 28.3% C, and 14.0% G. The PCGs utilized the standard mitochondrial start codon ATN (10 with ATG, 3 with ATA) and the regular stop codons (TAA or TAG) except for *cytb* (AGA). The incomplete stop codon was observed in *Nd4* (T–) and *coxIII* (TA–).

Phylogenetic analysis of the *H. pendleburyi* mitogenome was performed based on 13 PCGs from 21 Chiroptera species and one outgroup (*Mus musculus musculus*). We concatenated sequences from 13 PCGs and performed multiple alignments using MUSCLE (Edgar 2004). Subsequently, amino acid replacement models were estimated using ModelTest-NG (Darriba et al. 2020) and a maximum-likelihood phylogenetic tree was constructed by RAxML-NG (Kozlov et al. 2019) with 1000 bootstrap replicates (Figure 1). The result showed that *H. pendleburyi* was well grouped with *H. armiger* within the Hipposideridae clade, which had Rhinolophidae as a sister clade. Our result is consistent with the previous study by Lei and Dong (2016).

**Figure 1.**
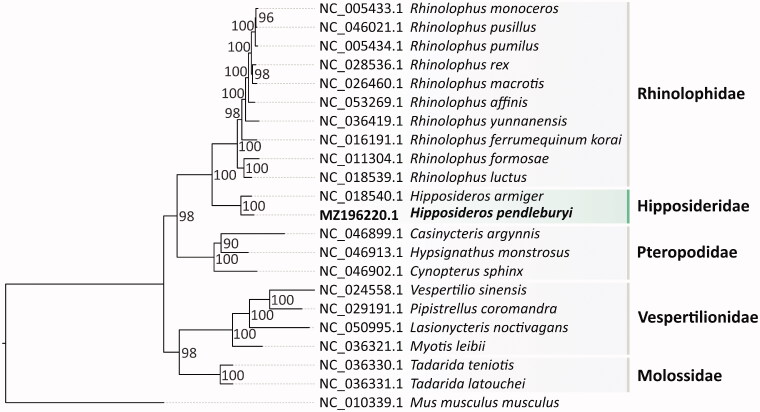
The phylogenetic relationships of *H. pendleburyi* and 21 Chiroptera species were inferred from maximum-likelihood analysis based on 13 PCGs. Numbers on branches represent bootstrap values.

## Data Availability

The genome sequence data that support the findings of this study are openly available in GenBank of NCBI at (https://www.ncbi.nlm.nih.gov/) under the accession no. MZ196220.1. The associated BioProject, SRA, and Bio-Sample numbers are PRJNA763996, SRR15927285, and SAMN21465651 respectively.
